# The First Silver-Based Plasmonic Nanomaterial for Shell-Isolated Nanoparticle-Enhanced Raman Spectroscopy with Magnetic Properties

**DOI:** 10.3390/molecules27103081

**Published:** 2022-05-11

**Authors:** Aleksandra Michałowska, Andrzej Kudelski

**Affiliations:** Faculty of Chemistry, University of Warsaw, 1 Pasteur Str., 02-097 Warsaw, Poland; a.michalowska10@uw.edu.pl

**Keywords:** magnetic–plasmonic nanostructures, surface-enhanced Raman scattering, coffee-ring effect

## Abstract

Nanostructures made of magnetic cores (from Fe_3_O_4_) with attached silver plasmonic nanostructures were covered with a very thin layer of silica. The (Fe_3_O_4_@Ag)@SiO_2_ magnetic–plasmonic nanomaterial can be manipulated using a magnetic field. For example, one can easily form homogeneous layers from this nanomaterial using a very simple procedure: deposition of a layer of a sol of such a nanostructure and evaporation of the solvent after placing the sample in a strong magnetic field. Due to the rapid magnetic immobilization of the magnetic–plasmonic nanomaterial on the investigated surface, no coffee-ring effect occurs during the evaporation of the solvent. In this contribution, we report the first example of a magnetic, silver-based plasmonic nanomaterial for shell-isolated nanoparticle-enhanced Raman spectroscopy (SHINERS). Nanoresonators based on silver plasmonic nanostructures locally enhance the intensity of the exciting electromagnetic radiation in a significantly broader frequency range than the previously used magnetic SHINERS nanoresonators with gold plasmonic nanostructures. Example applications where the resulting nanomaterial was used for the SHINERS investigation of a monolayer of mercaptobenzoic acid chemisorbed on platinum, and for a standard SERS determination of dopamine, are also presented.

## 1. Introduction

Measurements of the Raman spectra of molecules that are in close proximity to plasmonic nanostructures make it possible to obtain an exceptionally intense Raman signal. For example, Michaels et al. reported achieving a cross-section for Raman scattering of 2 × 10^−14^ cm^2^ per molecule [1], whereas the typical cross-section for Raman scattering is about 10^−^^29^ cm^2^ per molecule [2]. Raman scattering with enhanced efficiency due to the influence of certain structures is known as surface-enhanced Raman scattering (SERS). Usually, the main source of the SERS enhancement is a large local increase in the intensity of the electric field in the proximity of the illuminated plasmonic nano-objects. In some cases, the increase in the efficiency of the Raman scattering in the SERS effect is so great that it is possible to record a good-quality SERS spectrum of even a single molecule [3,4]. This means that Raman spectroscopy is one of the most sensitive analytical tools available.

Although the strongest SERS enhancement factor is observed for molecules that interact directly with the plasmonic metal nanostructures, in some cases the surface of the metallic plasmonic nanoresonator is covered with a layer of an inert material (e.g., SiO_2_, TiO_2_, ZrO_2_, or Al_2_O_3_) of a few nanometers in thickness. Such a thin protective layer does not significantly decrease the enhancement of the electromagnetic field generated by the plasmonic nanostructures, but at the same time does separate the metal from direct contact with the analyzed sample. The deposition of a protective layer on the surface of a metallic nanoresonator is especially important in investigations of biological structures such as proteins, because when certain biological structures interact with a metal surface, a large disturbance of their structure is observed—frequently, for example, protein denaturation [5,6]. Moreover, the deposition of an oxide layer on the surface of an electromagnetic nanoresonator often significantly increases the resonator’s stability and decreases its tendency to agglomerate. The Raman technique utilizing plasmonic nanoparticles with a deposited protective layer is known as SHINERS (an acronym for ‘shell-isolated nanoparticle-enhanced Raman spectroscopy’) [7].

In some cases, an interesting improvement of SERS nanoresonators is the introduction of strong magnetic properties in such structures, which makes it easy to manipulate SERS nanomaterials by means of a strong magnetic field. Such modification allows for simple concentration of nanostructures from their sol [8,9,10,11,12,13,14,15], or their easy and homogeneous deposition on an analyzed surface [16,17,18,19] by means of the following simple wet deposition method: (i) the deposition of a layer of a sol of the SERS nanoresonators with magnetic properties followed by (ii) the evaporation of the solvent while the sample is placed in a strong magnetic field [16,17,18]. When placed in a strong magnetic field, the magnetic–plasmonic SERS nanoresonators are quickly ‘magnetically glued’ to the analyzed surface, and therefore they do not migrate to the boundary of the drying region. This means that nanomaterials for SERS measurements that also have strong magnetic properties can be easily deposited on an analyzed surface without the occurrence of the ‘coffee-ring’ effect.

There have already been two literature reports on magnetic nanomaterials for SHINERS measurements in the form of gold nanoparticles protected with Fe_3_O_4_ (Au@Fe_3_O_4_) [16] and in the form of silica-covered nanostructures composed of magnetic Fe_3_O_4_ cores with gold nanostructures attached ((Fe_3_O_4_@Au)@SiO_2_) [17]. In this contribution, we report on a modification of previously developed (Fe_3_O_4_@Au)@SiO_2_ SHINERS nanoresonators using silver plasmonic nanostructures instead of gold ones. Silver provides a stronger plasmon resonance than gold, and so the SERS enhancement factors on silver can be significantly higher than those on gold [20]. Moreover, the large SERS enhancement factor on silver nanostructures may be achieved for any visible electromagnetic radiation, whereas when gold nanoparticles are used, large SERS enhancement factors can only be obtained for excitation radiation from the red part of the spectrum [20]. This means that using silver plasmonic nanostructures permits the easier combination of SHINERS with ‘standard’ resonance Raman scattering. Example applications of the (Fe_3_O_4_@Ag)@SiO_2_ nanomaterial for SHINERS investigation of a monolayer of para-mercaptobenzoic acid chemisorbed on platinum, and for the standard SERS determination of dopamine, are also presented.

## 2. Results and Discussion

### 2.1. Structural and Optical Characterization

**Figure 1:**
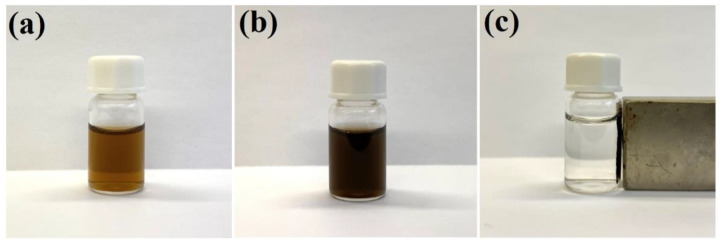
Photographs of: (**a**) the sol of silver nanoparticles (before mixing with the sol of Fe_3_O_4_ nanoparticles), (**b**) the mixture of the sol of Ag nanoparticles and the sol of Fe_3_O_4_ nanoparticles after sonication and shaking for 45 min, and (**c**) the sol of (Fe_3_O_4_@Ag)@SiO_2_ nanostructures after application of a strong external magnetic field. The used vessel has a volume of 2 mL. The sols were not diluted before the photo was taken.

As described in our previous work [17], gold nanostructures can be easily attached to the surface of polyethylenimine-modified Fe_3_O_4_ nanoparticles. We found that a similar attachment can also be realized using silver nanoparticles—in this case, the bonding is due to the formation of strong coordination bonds between the surface silver atoms and the nitrogen amine atoms in the polyethylenimine. The efficiency of the attachment of silver nanoparticles to the magnetic Fe_3_O_4_ cores was checked by applying a strong external magnetic field to the sonicated and shaken mixture of sols of Fe_3_O_4_ and Ag nanoparticles in a glass vessel. When a strong permanent magnet was attached to a vessel containing the sonicated and shaken mixture of Ag and polyethylenimine-modified Fe_3_O_4_ nanoparticles, the solution from which the formed nanocomposite was magnetically concentrated became practically transparent. This means that almost all of the strongly colored plasmonic silver nanoparticles were removed under the influence of the magnetic field, and that practically all of the silver nanoparticles became attached to the magnetic structures. The deposition of a layer of silica did not result in a noticeable separation of the Ag nanoparticles from the magnetic Fe_3_O_4_ structures—this nanomaterial also displays strong magnetic properties, and can be manipulated with a magnetic field—see Figure 1.

**Figure 2:**
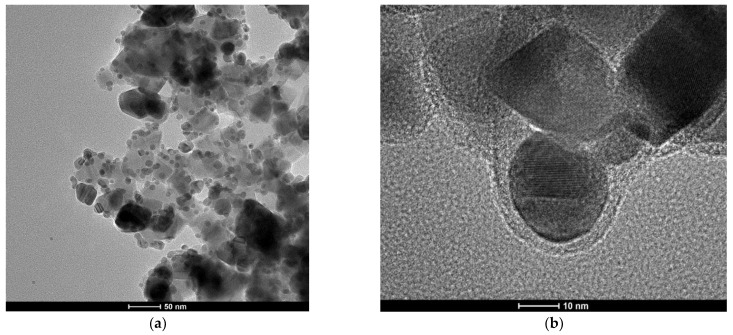
Example TEM images of the resulting (Fe_3_O_4_@Ag)@SiO_2_ nanocomposite. (**a**) 50 nm scale; (**b**) 10 nm scale. The accelerating voltage of the TEM microscope: 200 kV. The nanoparticles were deposited using a simple wet deposition method onto a 400-mesh copper grid covered with a carbon film.

The formation of (Fe_3_O_4_@Ag)@SiO_2_ nanostructures was proven by the TEM investigations. Figure 2 shows TEM images of the nanocomposite formed at different magnifications. As can be seen from Figure 2a, all of the silver nanostructures (visible as darker structures) were attached to larger Fe_3_O_4_ nanoparticles (with an average size of about 40 nm). As can be seen from the image obtained at a higher magnification (see Figure 2b), the composite formed was covered with an ultrathin layer of silica, the average thickness of which was about 3 ± 2 nm (see Figure 2).

**Figure 3:**
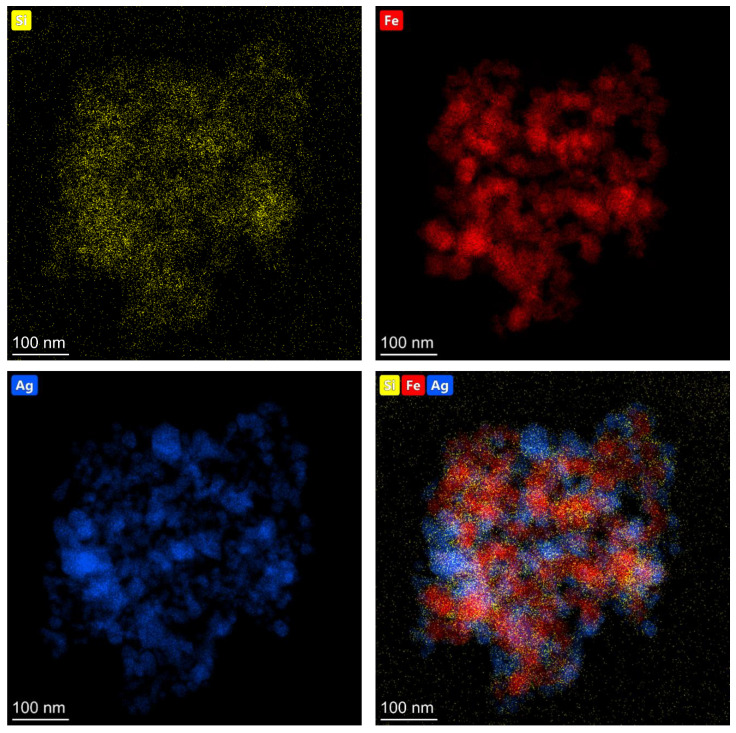
EDS maps showing the topographic distributions of silicon (yellow dots), iron (red dots), and silver (blue dots) in the resulting (Fe_3_O_4_@Ag)@SiO_2_ nanocomposite.

The elemental composition of the obtained (Fe_3_O_4_@Ag)@SiO_2_ nanomaterial was analyzed using energy dispersive X-ray spectroscopy (EDS), and the example EDS maps showing the distributions of various elements are shown in Figure 3.

**Figure 4:**
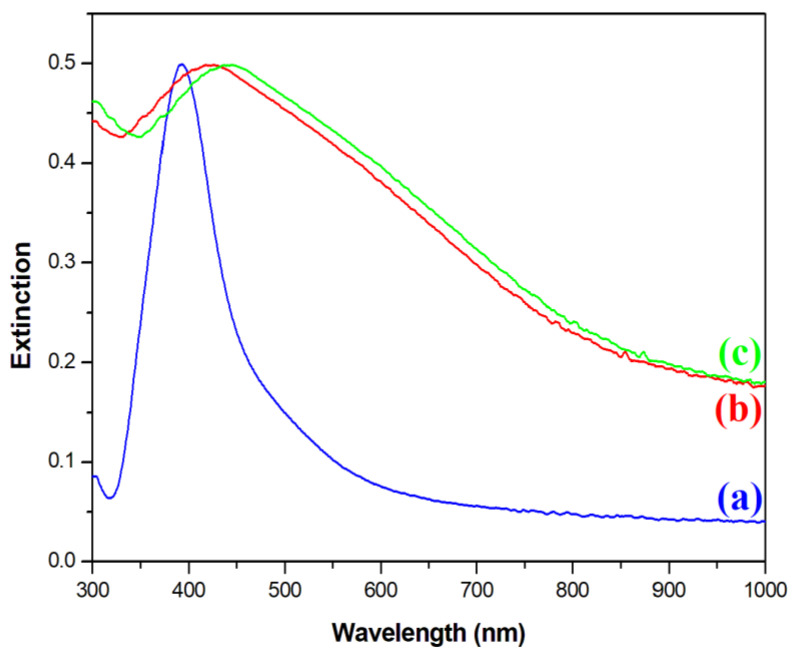
UV–vis extinction spectra of the sols of: (a) the Ag nanoparticles, (b) the Fe_3_O_4_@Ag nanocomposite before being covered with silica, and (c) the Fe_3_O_4_@Ag nanocomposite after being covered with a silica layer. The measurements were carried out using a standard rectangular 3.5 mL cuvette with an optical path of 1 cm.

Next, an optical characterization of the nanomaterials was performed. Figure 4 shows extinction spectra of the sols of synthesized Ag, Fe_3_O_4_@Ag, and (Fe_3_O_4_@Ag)@SiO_2_ nanoparticles. In the extinction spectrum of the sol of silver nanoparticles used in this work, a plasmonic peak at a wavelength of about 395 nm was clearly visible (see Figure 4). After the attachment of silver nanoparticles to the Fe_3_O_4_ cores, the plasmonic band significantly increased its width and red-shifted to 424 nm. Such a red-shift and broadening of the plasmonic band is a typical sign of the agglomeration of plasmonic nanoparticles [21]. This result of the UV–vis measurements was consistent with the TEM images obtained, where it was observed that several Ag nanoparticles were attached to almost all the Fe_3_O_4_ nanoparticles. The deposition of a SiO_2_ layer on the Fe_3_O_4_@Ag composite induced a further red-shift of the position of the plasmonic band to 437 nm. This red-shift was due to an increase in the refractive index in the visible range of the medium surrounding the plasmonic Ag structures [22] from about 1.33 for water [23] to about 1.46 for silica [24].

### 2.2. Applications of the Obtained Nanomaterials in the Raman Characterization of Monolayers of Para-Mercaptobenzoic Acid on Platinum

**Figure 5:**
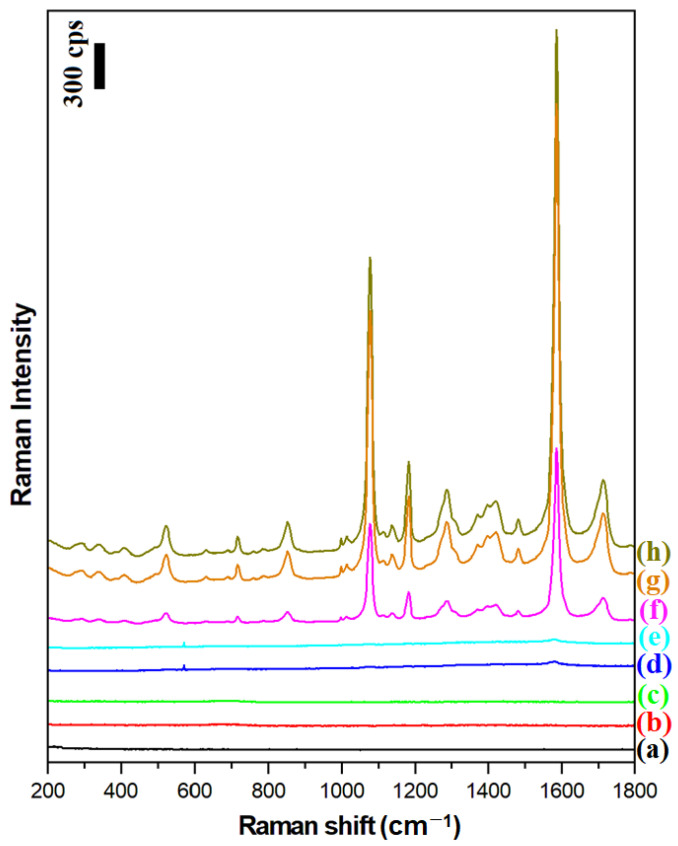
Raman spectra of: (a) the Pt/p-MBA monolayer before the deposition of the magnetic–plasmonic nanoresonators, (b) the Fe_3_O_4_@Ag nanoparticles deposited on the flame-annealed platinum plate, (c) the (Fe_3_O_4_@Ag)@SiO_2_ nanoparticles deposited on the flame-annealed platinum plate, (d) the Pt/p-MBA monolayer covered with Fe_3_O_4_@Au nanoparticles, (e) the Pt/p-MBA monolayer covered with (Fe_3_O_4_@Au)@SiO_2_ nanoparticles, (f) the Pt/p-MBA monolayer covered with Ag nanoparticles, (g) the Pt/p-MBA monolayer covered with (Fe_3_O_4_@Ag)@SiO_2_ nanoparticles, and (h) the Pt/p-MBA monolayer covered with Fe_3_O_4_@Ag nanoparticles. The wavelength of the excitation radiation: 532 nm. The power of the laser beam on the laser head: 70 mW. The accumulation time for a single Raman spectrum: 10 s.

The magnetic–plasmonic systems produced were used to perform SERS and SHINERS measurements of a model surface, namely a monolayer of para-mercaptobenzoic acid on platinum (Pt/p-MBA). As described previously, Pt/p-MBA monolayers can be easily formed in a very repeatable way by soaking a flame-annealed platinum plate for about one day in a saturated aqueous solution of para-mercaptobenzoic acid [16]. Figure 5 shows the Raman spectra of a Pt/p-MBA monolayer before the deposition of the plasmonic nanoresonators, and of such monolayers covered with Ag, Fe_3_O_4_@Ag, and (Fe_3_O_4_@Ag)@SiO_2_ nanomaterials. For comparison, the same figure also shows the Raman spectra of Pt/p-MBA monolayers covered with previously developed Fe_3_O_4_@Au and (Fe_3_O_4_@Au)@SiO_2_ nanoresonators [17], as well as the Raman spectra of Fe_3_O_4_@Ag and (Fe_3_O_4_@Ag)@SiO_2_ nanoresonators deposited on the surface of the flame-annealed platinum plate. From these spectra, one can conclude that:

1. The deposition of silver plasmonic nanostructures (Ag, Fe_3_O_4_@Ag, or (Fe_3_O_4_@Ag)@SiO_2_) induces a very large increase (by about 3 orders of magnitude) in the intensity of the recorded Raman spectra of p-MBA (compare spectrum a with spectra f, g, h);
2. The previously developed Fe_3_O_4_@Au and (Fe_3_O_4_@Au)@SiO_2_ nanoresonators do not effectively enhance the Raman spectra when green excitation radiation (λ_exc_ = 532 nm) is used—see spectra d and e. This means that the previously developed magnetic–plasmonic nanoresonators containing Au nanostructures do not work with this range of excitation radiation;
3. The Raman spectral background generated by the Fe_3_O_4_@Ag and (Fe_3_O_4_@Ag)@SiO_2_ nanoresonators on their own is very weak—see spectra b and c—and does not disturb the standard SERS measurements;
4. The deposition of a very thin (approximately 3 nm) silica layer on the surface of the Fe_3_O_4_@Ag nanostructures induces only a very small (7% on average) decrease in the intensity of the measured Raman spectrum—compare spectra g and h;
5. A much higher intensity of the Raman spectrum of p-MBA is observed when silver nanoparticles (nanoresonators) are attached to the Fe_3_O_4_ nanostructures than when they are not immobilized—compare the intensity of spectrum f with those of spectra g and h. This effect is due to the formation of arrangements among the immobilized plasmonic nanoparticles in close proximity to each other. Here, a more efficient coupling of plasmons in nearby nanoparticles occurs, leading to the formation of many SERS hot spots between the silver nanostructures (agglomerates of plasmonic nanoparticles generate a significantly stronger SERS signal than nonaggregated plasmonic nanoparticles [25,26]).

The two strongest bands in the SERS spectrum of the p-MBA, at 1077 cm^−1^ and 1587 cm^−1^, are due to the ν_12_ and ν_8a_ vibrations of the aromatic ring of p-MBA, respectively [27]. The third most intense band at 1185 cm^−1^ is due to the δ(C-H) vibration [27].

### 2.3. The Influence of a Magnetic Field on the Morphology of Layers Formed from a Magnetic–Plasmonic (Fe_3_O_4_@Ag)@SiO_2_ Nanocomposite

**Figure 6:**
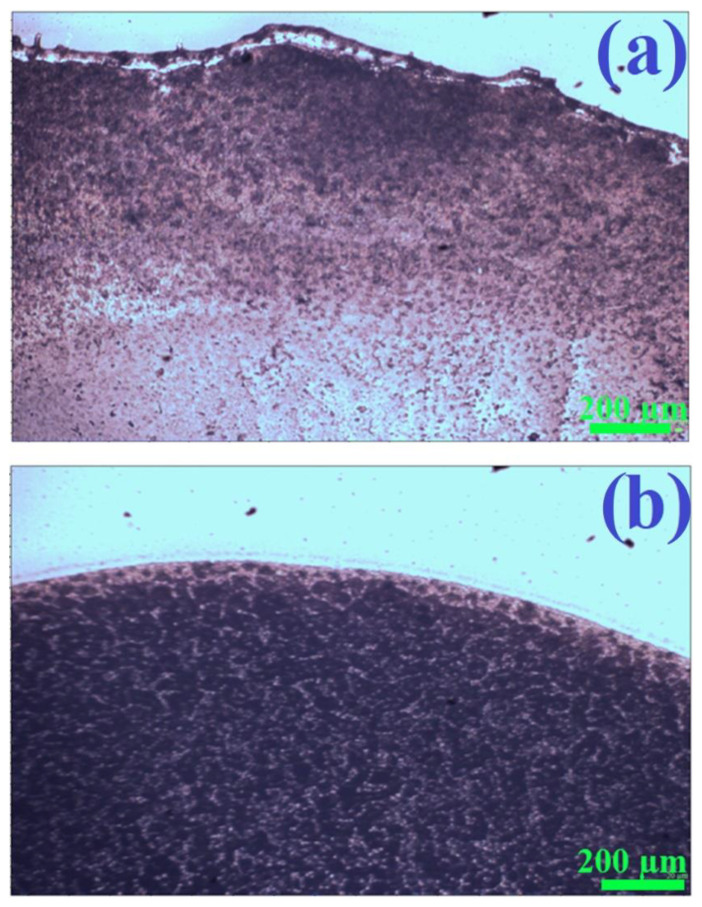
Photographs of fragments of films of (Fe_3_O_4_@Ag)@SiO_2_ nanoparticles on a glass substrate obtained by drying a drop of the sol of (Fe_3_O_4_@Ag)@SiO_2_ nanoparticles: (**a**) without applying a strong external magnetic field, and (**b**) when the sample was placed in a strong external magnetic field. For the photographs presented, brightness correction was performed automatically—the physical sense is related only to the differences in the brightness of different areas of the photo. Images obtained using an Olympus BX40 microscope with a 4× objective.

As mentioned in the introduction, plasmonic nanostructures with magnetic properties can easily be homogeneously deposited on the surfaces analyzed herein using a simple method known as wet deposition: dropping a sol containing magnetic nanoresonators on the surface under investigation, and then evaporating the solvent with the sample placed in a strong magnetic field (‘magnetic immobilization’ prevents the movement of nanostructures to the edges of the evaporated area, thereby eliminating the coffee-ring effect). We found that the magnetic properties of the (Fe_3_O_4_@Ag)@SiO_2_ nanoparticles formed were strong enough to eliminate the coffee-ring effect when drying a drop of a sol of (Fe_3_O_4_@Ag)@SiO_2_ nanoparticles in a strong magnetic field. Figure 6 shows the visual appearance of a glass substrate covered with films of a (Fe_3_O_4_@Ag)@SiO_2_ nanomaterial, dried with and without the application of a strong external magnetic field. As can be seen, when the deposited layer of sol was dried without a magnetic field, there was an accumulation of nanoparticles at the boundary of the drying region. In contrast, when the solvent was evaporated with the glass substrate in the presence of a strong external magnetic field, the distribution of deposited nanomaterial was homogeneous, with no accumulation of nanoparticles is any particular area.

**Figure 7:**
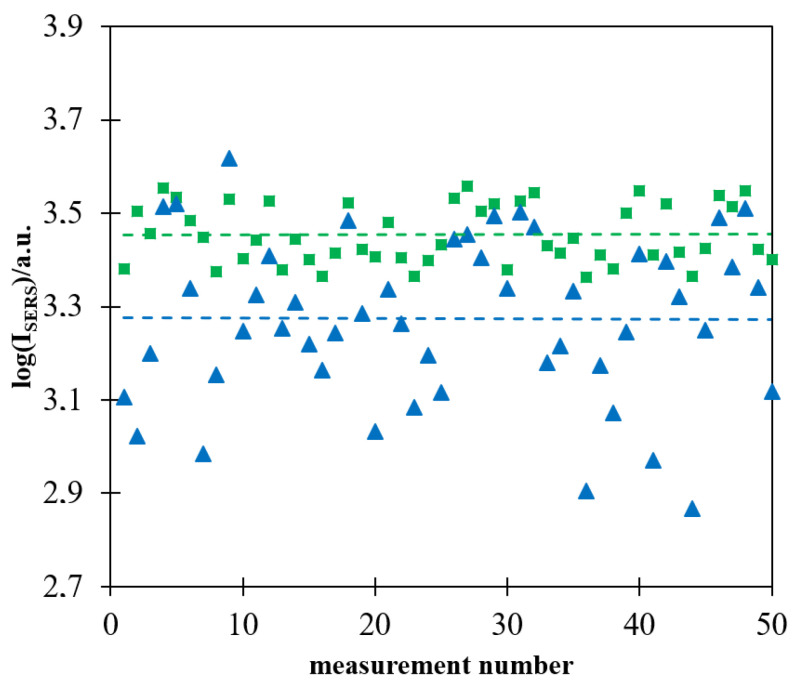
Logarithm of the intensity of the strongest Raman band at 1587 cm^−1^ in the spectrum of the Pt/p-MBA monolayer covered by the (Fe_3_O_4_@Ag)@SiO_2_ nanomaterial in each of 50 subsequent measurements: (■) evaporation of the solvent performed when the sample was placed in a strong magnetic field, (▲) evaporation of the solvent performed without application of a strong magnetic field. The dashed lines show the average values calculated from 50 measurements. The wavelength of the excitation radiation: 532 nm. The power of the laser beam on the laser head: 70 mW. The accumulation time for a single Raman spectrum: 10 s.

As already reported in some previous publications, the intensity of the Raman spectrum recorded in a single measurement using a more homogeneously deposited layer of magnetic–plasmonic nanostructures is significantly more reproducible [16,17]. Figure 7 shows a logarithm of the intensity of the strongest Raman band at 1587 cm^−^^1^ in 50 subsequently measured Raman spectra of a Pt/p-MBA monolayer covered with (Fe_3_O_4_@Ag)@SiO_2_ nanoparticles. The measurements were carried out for films of (Fe_3_O_4_@Ag)@SiO_2_ nanoparticles prepared with and without the application of a strong external magnetic field during evaporation of the solvent. As in the case of the previously formed magnetic–plasmonic materials for SHINERS measurements [16,17], the deviation from the mean value was significantly lower for films prepared when a magnetic field was used during the drying process. It is likely that the main reason for a certain nonreproducibility of the measured intensity of the SERS spectra of p-MBA, observed even in the case of measurements carried out on (Fe_3_O_4_@Ag)@SiO_2_ layers prepared with the application of a strong external magnetic field during evaporation of the solvent, was a partial agglomeration of magnetic–plasmonic structures, the occurrence of which was facilitated by the magnetic properties of the nanomaterial used.

### 2.4. The Stability of Magnetic–Plasmonic Nanocomposites after Deposition of a Silica Layer

**Figure 8:**
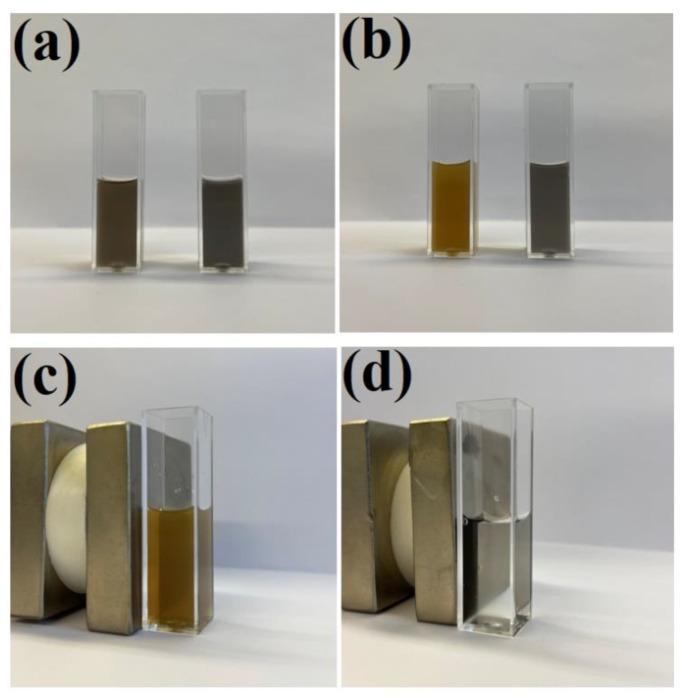
Photographs of sols of: (**a**) the Fe_3_O_4_@Ag (left) and (Fe_3_O_4_@Ag)@SiO_2_ (right) before the addition of hydrochloric acid, (**b**) the Fe_3_O_4_@Ag (left) and (Fe_3_O_4_@Ag)@SiO_2_ (right) one hour after the addition of hydrochloric acid to a final concentration of 0.1 M, (**c**) the Fe_3_O_4_@Ag one hour after the addition of hydrochloric acid—the cuvette with the sol is placed near a strong neodymium magnet—and (**d**) the (Fe_3_O_4_@Ag)@SiO_2_ one hour after the addition of hydrochloric acid—the cuvette with the sol is placed near a strong neodymium magnet. The cuvette used was a standard square 3.5 mL cuvette with an optical path of 1 cm. The sols were not diluted before the photo was taken.

As described in the introduction, the deposition of a silica layer on some nanomaterials used for SERS measurements significantly increases their stability. The same effect is observed after the deposition of a silica layer on Fe_3_O_4_@Ag nanostructures. For example, when some amount of hydrochloric acid is introduced to a sol of Fe_3_O_4_@Ag nanoparticles, it can be observed that the Fe_3_O_4_ begins to dissolve (after some time, the material is no longer magnetically accumulated in such samples). However, when a magnetic–plasmonic Fe_3_O_4_@Ag nanocomposite was covered with even a very thin layer of SiO_2_ (about 3 nm thick), its chemical stability significantly increased and no signs of chemical decomposition were visible even after soaking the (Fe_3_O_4_@Ag)@SiO_2_ nanostructures in a 0.1 M HCl solution for 1 h—see Figure 8. However, when the contact of the (Fe_3_O_4_@Ag)@SiO_2_ nanostructures with a 0.1 M HCl solution was significantly longer (e.g., 1 day), decomposition of the (Fe_3_O_4_@Ag)@SiO_2_ nanomaterial was observed. When using a more diluted (0.01 M) solution of HCl or a 0.1 M solution of citric acid, no significant signs of chemical decomposition of (Fe_3_O_4_@Ag)@SiO_2_ were visible even after 1 day (the material retained its magnetic properties).

### 2.5. An Example Application of (Fe_3_O_4_@Ag)@SiO_2_ Nanoparticles in the Raman Detection of Dopamine

**Figure 9:**
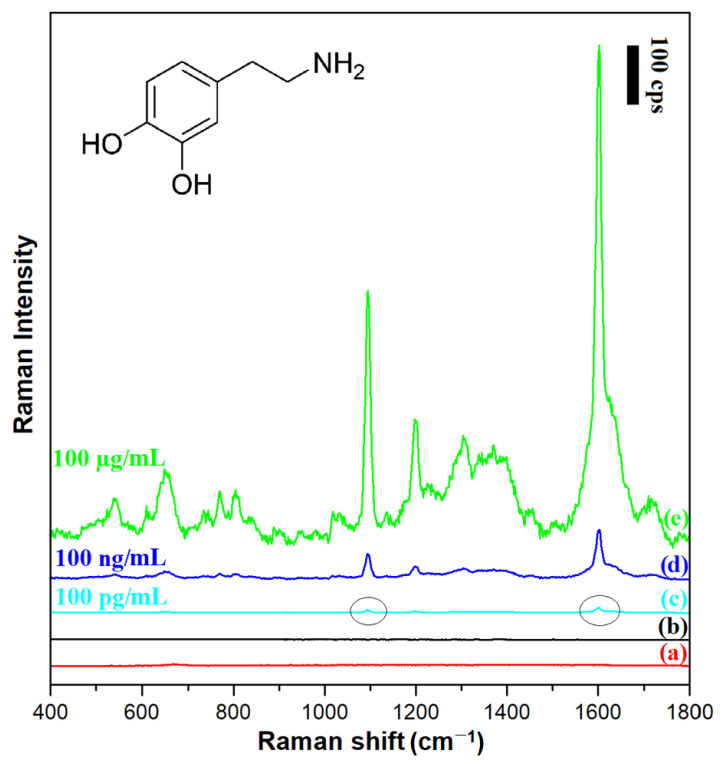
Raman spectra of: (a) the film of (Fe_3_O_4_@Ag)@SiO_2_ nanoresonators deposited on a glass substrate, (b) the layer of 100 µg/mL dopamine solution on a glass substrate (without a film of nanoresonators), (c) the film of (Fe_3_O_4_@Ag)@SiO_2_ nanoresonators deposited on a glass substrate in contact with a 100 pg/mL dopamine solution, (d) the film of (Fe_3_O_4_@Ag)@SiO_2_ nanoresonators deposited on a glass substrate in contact with a 100 ng/mL dopamine solution, (e) the film of (Fe_3_O_4_@Ag)@SiO_2_ nanoresonators deposited on a glass substrate in contact with a 100 µg/mL dopamine solution. The wavelength of the excitation radiation: 532 nm. The power of the laser beam on the laser head: 70 mW. The accumulation time for a single Raman spectrum: 30 s.

Dopamine (3,4-dihydroxyphenylethylamine) is one of the most important neurotransmitters (from the catecholamine group) in the human body (the inset in Figure 9 shows the structural formula of dopamine). Dopamine operates in the hypothalamus and the pituitary gland, and directly affects a person’s emotions [28]. Abnormal dopamine levels are associated with neurological disorders such as schizophrenia, Parkinson’s disease, depression, and Huntington’s disease [29,30]. In this work, we decided to use dopamine as a Raman scatterer to verify whether a silica-covered magnetic–plasmonic SERS substrate could be used to detect dopamine when its concentration in the surrounding liquid was within the range of physiological concentrations.

Figure 9 shows SERS spectra of dopamine chemisorbed from solutions with different concentrations on a layer of (Fe_3_O_4_@Ag)@SiO_2_ nanoparticles. The figure also shows, for comparison, the normal Raman spectrum of a 100 µg/mL dopamine solution, as well as the spectral background generated by the (Fe_3_O_4_@Ag)@SiO_2_ nanoresonators themselves. It was evident a reliable SERS spectrum of dopamine could be obtained using a silica-protected SERS substrate even when the concentration of dopamine in the surrounding solution was 100 pg/mL, that is, at the level of the physiological concentration of dopamine in many body fluids [31]. According to the criterion of a signal-to-noise (S/N) ratio of 3/1, the limit of detection (LOD) of dopamine was calculated to be 2.3 × 10^−10^ mol/L. This means that LOD of dopamine achieved in these SERS experiments was similar to that achieved by Zhang et al. in very sensitive photoluminescence analysis (3 × 10^−10^ mol/L [32]), and was significantly lower than LODs achieved in electrochemical detections (for example: 1 × 10^−8^ mol/L when porphyrin-functionalized graphene was used [33] or 1.1 × 10^−8^ mol/L when niobium microelectrodes coated with carbon nanotubes were used [34]). Dopamine is often administered in a 0.9% NaCl solution (saline). We found that addition of this amount of NaCl to the analyzed solution did not increase the LOD of dopamine by the SERS method. The recorded dopamine spectrum is dominated by a band at 1607 cm^−^^1^ due to the ring deformation vibration [35]. The other Raman bands visible in the spectrum are probably due to: the CH in-plane ring deformation vibration (567 cm^−^^1^), the aliphatic chain C–C vibration (639 cm^−^^1^), the CH out-of-plane vibrations (at 767 cm^−^^1^ and 802 cm^−^^1^), the C-C-N stretching vibration (1095 cm^−^^1^), the CO stretching vibration (1206 cm^−^^1^), the CH wagging vibration (1387 cm^−^^1^), and the ring deformation vibration (1607 cm^−^^1^) [35].

## 3. Materials and Methods

### 3.1. Materials

Iron(II) sulfate heptahydrate, silver nitrate, tetraethyl orthosilicate, sodium borohydride, sodium hydroxide, potassium nitrate, polyethylenimine (with an average molecular weight of 40,000), trisodium citrate dihydrate, 4−mercaptobenzoic acid, and dopamine hydrochloride were acquired from Sigma-Aldrich. A 25% aqueous solution of ammonia, sodium chloride, citric acid and isopropyl alcohol were purchased from POCH S.A. All of the reagents mentioned above were used without further purification. High-purity nitrogen (≥99.999%) was acquired from Air Products. The water used in all the experiments was purified using a Millipore Milli-Q system (Merck Millipore, Burlington, MA, USA). Except for the process of deposition of a SiO_2_ layer, all of the other reactions were carried out in aqueous solutions.

### 3.2. Synthesis of Polyethylenimine-Stabilized Fe_3_O_4_ Nanoparticles

Polyethylenimine-stabilized Fe_3_O_4_ nanoparticles were synthetized according to a slightly modified procedure developed by Kwizera et al. [36]. Briefly, 20 mL of water was first deaerated by bubbling with nitrogen. Next, 0.175 g of FeSO_4_⸱7H_2_O, 2.5 mL of a 2.0 M KNO_3_, 2.5 mL of a 1.0 M NaOH, and 5 mL of an 8 mg/mL polyethylenimine solution were added subsequently. The resulting mixture was kept under a nitrogen flow for 2 h at 90 °C. In the next step, the obtained iron oxide precursor nanoparticles were concentrated using a strong magnetic field, and then rinsed with water and again dispersed in water. The sol of purified iron oxide precursor nanoparticles was kept in contact with air for at least 1 day at 4 °C. X-ray diffraction and Raman analyses of the material obtained as a result of this procedure were described in detail in our previous work, and showed that the outermost parts of the formed iron oxide nanostructures consisted practically only of Fe_3_O_4_ [17].

### 3.3. Synthesis of Silver Nanoparticles and Their Attachment to the Iron Oxide Cores

**Figure 10:**
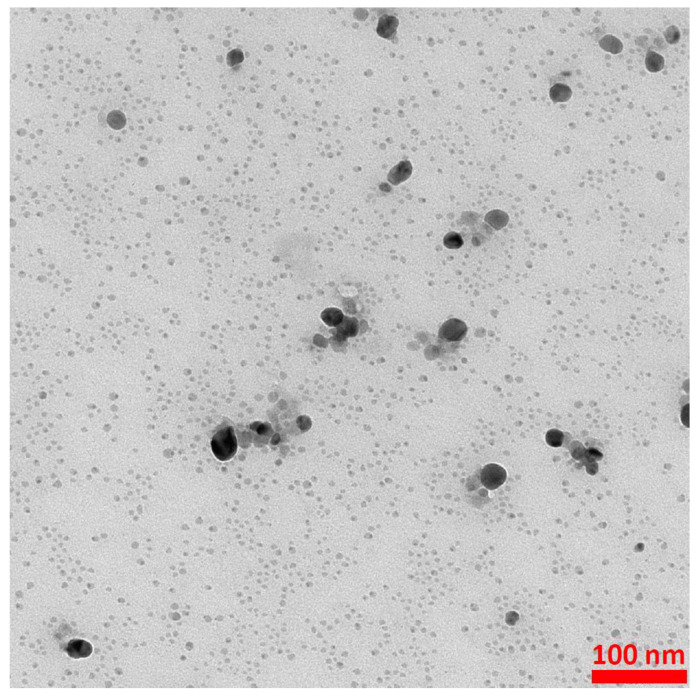
Example TEM image of the formed Ag nanoparticles. The accelerating voltage of TEM microscope: 200 kV. The nanoparticles were deposited using a simple wet deposition method onto 400-mesh copper grid covered with a carbon film.

In order to obtain silver nanoparticles, 0.2 mL of a 1 mM AgNO_3_ solution and 0.9 mL of a 3 mM sodium citrate solution were added to 50 mL of water. The resulting mixture was stirred vigorously, and then 1 mL of a freshly prepared 0.03 M NaBH_4_ solution was slowly added dropwise while stirring. Vigorous stirring was continued for 30 min after the dropwise addition of NaBH_4_ was completed. A TEM image of the silver nanoparticles obtained is presented in Figure 10. As can be seen from Figure 10, the vast majority of the formed Ag nanoparticles were below 10 nm, and only a very small fraction of the nanoparticles were much larger (10–30 nm).

Next, the attachment of silver nanoparticles to the polyethyleneimine-modified Fe_3_O_4_ nanoparticles was carried out. For this purpose, 8 mL of a sol of silver nanoparticles and 400 µL of a sol of Fe_3_O_4_ nanoparticles modified with polyethyleneimine were introduced into a glass reactor, and the reaction mixture was sonicated for 20 min and then shaken for another 45 min. When a strong permanent magnet was attached to the vessel containing the mixture (after sonication and shaking), the solution from which the formed nanocomposite was magnetically concentrated became practically transparent. This means that almost all of the silver nanoparticles were removed under the influence of the magnetic field, and it was therefore concluded that practically all the plasmonic silver nanoparticles became attached to the magnetic cores.

In a similar manner, gold nanoparticles attached to polyethyleneimine-modified Fe_3_O_4_ nanoparticles were also produced for use as a reference material (for details, see [17]).

### 3.4. Deposition of a Silica Layer

In order to cover the nanocomposite with a protective layer of silica, the Stober method of SiO_2_ deposition, described in detail in our previous work [17], was used. Briefly, 1.0 mL of a 5.5-fold concentrated (in comparison with the initial solution—for details, see Section 3.3) sol of Fe_3_O_4_@Ag nanostructures was added under continuous stirring to a solution obtained by mixing 8 mL of isopropyl alcohol, 0.2 mL of a 25% aqueous solution of ammonia, and 2 μL of tetraethyl orthosilicate while the solution was maintained at 27 °C. The stirring of the reaction mixture was continued at 27 °C for 15 min. The (Fe_3_O_4_@Ag)@SiO_2_ nanostructures formed were then collected using magnetic separation and washed twice with water.

In a similar manner, (Fe_3_O_4_@Au)@SiO_2_ nanostructures were also produced for use as a reference material (for details, see [17]).

### 3.5. Experimental Techniques

The surface morphology of the obtained nanomaterials was determined using a Talos F200X transmission electron microscope (TEM) working at an accelerating voltage of 200 kV. The elemental composition of the (Fe_3_O_4_@Au)@SiO_2_ samples were analyzed using a Brucker BD4 energy dispersive X-ray spectroscopy (EDS) instrument.

UV–vis extinction spectroscopic measurements were performed using a Thermo Scientific Evolution 201 spectrophotometer.

The Raman measurements were carried out using a Horiba Jobin-Yvon Labram HR800 spectrometer equipped with a Peltier cooled charge-coupled device detector (1024 × 256 pixels), a 600 groove/mm holographic grating, and an Olympus BX40 microscope with a long-distance 50× objective. A frequency-doubled Nd:YAG laser provided the excitation radiation at a wavelength of 532 nm.

## 4. Conclusions

A surface-protected magnetic–plasmonic nanomaterial based on silver nanostructures was applied for the first time for shell-isolated nanoparticle-enhanced Raman spectroscopy (SHINERS) measurements. Silver nanoresonators effectively enhanced the intensity of the Raman spectrum over a significantly broader frequency range of excitation radiation than the previously used magnetic SHINERS nanoresonators based on gold plasmonic nanostructures. The deposition of even a very thin silica layer (with an average thickness of about 3 nm) on the surface of Fe_3_O_4_@Ag nanostructures significantly increased their chemical resistance. The resulting (Fe_3_O_4_@Ag)@SiO_2_ nanomaterial has strong magnetic properties, such that it can easily be manipulated by a magnetic field; for example, one can easily form homogeneous layers of this material using a very simple procedure: the deposition of a layer of a sol of such nanostructures followed by evaporation of the solvent in the presence of a strong magnetic field. Due to the quick ‘magnetic gluing’ of the nanomaterial in the magnetic field, the coffee-ring effect is practically eliminated (no transport of nanoparticles to the boundary of the drying area was observed). The layers of magnetic–plasmonic nanostructures formed when the drying was carried out on a sample placed in a strong magnetic field exhibited a significantly lower deviation from the mean value of the intensity of the measured SHINERS spectra.

In addition to the model SHINERS measurements, the surface-protected (Fe_3_O_4_@Ag)@SiO_2_ nanomaterial obtained was also tested in standard SERS measurements of dopamine. We showed that a reliable SERS spectrum of dopamine can be obtained using this silica-protected SERS substrate even when the concentration of dopamine in the surrounding solution is 100 pg/mL, that is, at the physiological concentration of dopamine in some body fluids.

## Data Availability

The data presented in this study are available on request from A.M.

## Sample Availability

Samples of the nanomaterials presented in this study are available on request from the authors.
